# The Impact of Systemic Inflammation on Recurrence in Patients with Congenital Nasolacrimal Duct Obstruction

**DOI:** 10.3390/jcm13226834

**Published:** 2024-11-13

**Authors:** Hüseyin Findik, Feyzahan Uzun, Muhammet Kaim, Mehmet Birinci, Metin Çeliker, Murat Okutucu, Mehmet Gökhan Aslan

**Affiliations:** 1 Department of Ophthalmology, School of Medicine, Recep Tayyip Erdogan University, 53100 Rize, Turkey; feyzahan.ekici@erdogan.edu.tr (F.U.); muhammet.kaim@erdogan.edu.tr (M.K.); murat.okutucu@erdogan.edu.tr (M.O.); mehmetgokhan.aslan@erdogan.edu.tr (M.G.A.); 2 Department of Otorhinolaryngology, School of Medicine, Recep Tayyip Erdogan University, 53100 Rize, Turkey; mehmet.birinci@erdogan.edu.tr (M.B.); metin.celiker@erdogan.edu.tr (M.Ç.)

**Keywords:** inflammation, intubation, lymphocytes, monocytes, nasolacrimal duct obstruction, neutrophils, platelets

## Abstract

**Background**/**Objective:** Congenital nasolacrimal duct obstruction (CNLDO) is commonly treated by probing, but recurrence remains a clinical issue. This study investigates the potential role of inflammatory biomarkers, such as the neutrophil-to-lymphocyte ratio (NLR), monocyte-to-lymphocyte ratio (MLR), and platelet-to-lymphocyte ratio (PLR), in predicting recurrence after probing in children with CNLDO. **Methods:** This retrospective cohort study included 172 patients who underwent initial probing for unilateral CNLDO. The patients were then categorized into two groups: those who experienced the complete resolution of symptoms after primary probing, and those who required Ritleng tube intubation due to recurrence following primary probing. Blood samples for calculating inflammatory biomarkers in all subjects were collected during general anesthesia preparation prior to initial probing. NLR, MLR, and PLR values were compared between the groups using the independent samples *t*-test. The predictive performance of the inflammatory biomarkers for recurrence was assessed using Receiver Operating Characteristic (ROC) curve analysis. **Results:** A total of 110 patients were included in the probing group, while 62 patients were in the recurrence group. The mean age at the time of the initial probing procedure was 15 ± 4.06 months in the probing group and 15.83 ± 4.02 months in the recurrence group. There was no difference in the duration of the probing procedure between the groups. The mean age at the time of Ritleng tube intubation in the recurrence group was 37.80 ± 13.34 months. The recurrence group exhibited significantly higher values in all analyzed inflammatory markers compared to the probing group, including the NLR (1.12 ± 0.56 vs. 0.86 ± 0.39, *p* = 0.002), MLR (0.16 ± 0.06 vs. 0.14 ± 0.06, *p* = 0.005), and PLR (95.13 ± 24.34 vs. 82.23 ± 22.77, *p* < 0.001). ROC curve analysis indicated that these inflammatory biomarkers demonstrated moderate performance in predicting recurrence. **Conclusions:** Recurrence following probing in children with CNLDO was associated with complete blood cell count-derived inflammatory biomarkers. The preoperative assessment of these biomarkers may aid in the individualization of disease management and inform the development of new therapeutic strategies.

## 1. Introduction

Congenital nasolacrimal duct obstruction (CNLDO) is a prevalent ophthalmologic condition affecting newborns and infants, characterized by the failure of tears to drain properly through the nasolacrimal duct into the nasal cavity, leading to symptoms such as epiphora and recurrent eye infections [1]. If left untreated, CNLDO can impair vision and hinder social development [2]. The primary etiology involves a failure of the Hasner valve at the distal end of the nasolacrimal duct to open, which obstructs normal tear drainage, resulting in persistent epiphora and mucopurulent discharge [3]. The pathophysiology of CNLDO encompasses complex interactions involving inflammation and immune responses [4]. Probing, one of the most widely utilized interventions for CNLDO, involves using a thin wire to open the obstructed duct and is typically performed within the first year of life, demonstrating a high success rate [5]. However, recurrence occurs in approximately 10–20% of cases, necessitating further, often more invasive, interventions such as the placement of a Ritleng tube [1,6,7].

Inflammation plays a significant role in CNLDO recurrence. Trauma and bleeding induced by probing may elicit an inflammatory response, contributing to re-occlusion of the duct [8]. The effective control of inflammation is therefore critical for successful treatment outcomes. The immune system, particularly the roles of lymphocytes, monocytes, platelets, and neutrophils, is central to the modulation of inflammation and tissue healing [9,10]. The dysregulation of these immune cells can lead to excessive inflammation and tissue damage, potentially exacerbating recurrence.

Recent studies have identified complete blood cell count-derived biomarkers such as the neutrophil-to-lymphocyte ratio (NLR), monocyte-to-lymphocyte ratio (MLR), and platelet-to-lymphocyte ratio (PLR) as valuable tools for assessing inflammation and healing processes [11]. In addition to markers such as the NLR, MLR, and PLR, other blood-derived inflammatory biomarkers, including the Systemic Inflammatory Index (SII), Systemic Inflammatory Response Index (SIRI), and Aggregate Inflammatory Systemic Index (AISI), have been investigated in relation to both the success and recurrence of various surgical procedures, as well as the treatment response and recurrence of cancers and various chronic systemic diseases [12,13]. These biomarkers offer insights into the severity of inflammation and the body’s healing capacity, with lymphocyte balance playing a pivotal role in controlling inflammation and promoting tissue repair [8,14]. Imbalances in lymphocyte levels may contribute to uncontrolled inflammation and tissue damage, which could be a key factor in the recurrence of CNLDO following probing.

This study aims to investigate the association between these inflammatory biomarkers and the recurrence of CNLDO after probing in children. By elucidating the relationship between biomarker levels and disease recurrence, the study seeks to underscore the importance of maintaining systemic immune balance in the management of CNLDO.

## 2. Materials and Methods

A retrospective review of medical charts was conducted for patients diagnosed with unilateral CNLDO at the Department of Ophthalmology, Recep Tayyip Erdogan University Training and Research Hospital, between January 2018 and December 2023, with approval obtained from the institutional ethics committee (IRB Number: E-64960800-799-257363134). The researchers adhered to the principles of the Declaration of Helsinki, and written informed consent was obtained from all participants prior to the surgical procedures.

**Figure 1:**
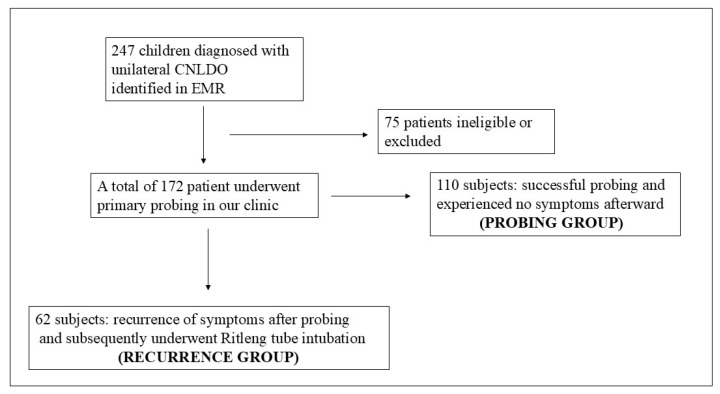
The flow chart of the patients with the diagnosis of CNLDO.

The study population was divided into two groups: (1) Probing Group: patients who underwent successful probing and experienced no symptoms afterward. (2) Recurrence Group: patients who experienced a recurrence of symptoms after initial probing and subsequently underwent Ritleng tube intubation. The patient flow throughout the study can be seen in Figure 1. Success or recurrence was defined as the absence or presence of epiphora, tear pooling, and mucopurulent discharge—three main characteristics of CNLDO—at least four weeks following probing [15]. In addition to these criteria, the dye disappearance test was also performed to confirm the presence of obstruction [15]. Inclusion criteria were patients aged 12 to 24 months with unilateral CNLDO who underwent primary probing. Exclusion criteria included patients with systemic inflammatory diseases, bilateral CNLDO, congenital anomalies, a history of previous ocular surgery, or inadequate follow-up of less than six months.

The demographic characteristics, medical history, clinical features including complete ophthalmologic and otolaryngologic findings, and laboratory test results of the patients were retrieved from electronic health records by trained researchers. A complete otolaryngologic examination was conducted both before the initial probing procedure for both groups and, in the recurrence group, prior to Ritleng tube intubation. Preoperative blood samples, routinely obtained after 6–8 h of fasting, were collected from all participants during the preparation for general anesthesia prior to the initial probing procedure; systemic inflammation markers were calculated from these samples. Complete blood counts were performed using hematology analyzers, and the results were recorded. The NLR, MLR, and PLR values were then calculated by dividing the neutrophil, monocyte, and platelet counts by the lymphocyte count, respectively.

### Statistical Analysis

The Kolmogorov–Smirnov test was used to assess the normality of continuous variables. Variables with *p* > 0.05 were considered normally distributed.

Descriptive Statistics: Continuous variables were expressed as the mean ± standard deviation and median, while categorical variables were presented as counts.

Group Comparisons: An independent samples *t*-test was used for normally distributed continuous variables, whereas the Mann–Whitney U test was applied to continuous variables that did not follow a normal distribution. The Pearson Chi-square test was employed for comparing categorical variables.

ROC Analysis: This analysis was conducted to assess the diagnostic performance of inflammatory biomarkers—namely, the NLR, MLR, and PLR—in predicting recurrence of CNLDO. The area under the curve (AUC) was calculated for each marker, accompanied by 95% confidence intervals. Optimal cut-off values were determined using the Youden index, and the analysis was repeated to comprehensively evaluate the predictive performance of these biomarkers, providing AUC, sensitivity, specificity, positive predictive value, negative predictive value, and optimal cut-off values for each.

Power Analysis: A post hoc power analysis was conducted using G*Power software (version 3.1.9.7, Heinrich Heine University, Düsseldorf, Germany). The sample size was calculated with a significance level of α = 0.05, a power of 85%, and an effect size of 0.5.

All statistical analyses were performed using IBM SPSS Statistics for Windows, Version 29.0 (IBM Corp., Armonk, NY, USA). A *p*-value of <0.05 was considered statistically significant.

## 3. Results

A total of 247 children diagnosed with unilateral CNLDO between January 2018 and December 2023 were identified through our electronic medical records. Of these, 75 patients were excluded due to missing data, inadequate follow-up time, or not meeting the inclusion criteria.

A total of 110 patients (53 males, 57 females) were included in the probing group, while 62 patients (28 males, 34 females) were in the recurrence group. There was no significant difference in gender distribution between the two groups (*p* = 0.41). The mean age at the time of the initial probing procedure was 15 ± 4.06 months in the probing group and 15.83 ± 4.02 months in the recurrence group. There was no difference in the duration of the probing procedure between the groups (*p* = 0.19). The mean age at the time of Ritleng tube intubation in the recurrence group was 37.80 ± 13.34 months. Additionally, no significant differences were found between the groups concerning the presence of comorbid conditions such as adenoid and tonsil hypertrophy, sinusitis, allergic rhinitis, or pharyngitis. All demographic characteristics of the patients are presented in Table 1.

Upon evaluating the complete blood cell count parameters, no statistically significant differences were observed between the groups regarding white blood cell, lymphocyte, monocyte, neutrophil, eosinophil, basophil, hemoglobin, and platelet counts (Table 2).

### 3.1. Analysis of Inflammatory Markers

Our study identified statistically significant differences in inflammatory biomarkers between the primary probing group and the recurrence group in patients with CNLDO (Table 2). The NLR values in the recurrence group (1.12 ± 0.56) were significantly higher than those in the probing group (0.86 ± 0.39) (*p* = 0.002). Additionally, elevated NLR levels may also be associated with an increased risk of recurrence. The MLR values were significantly higher in the recurrence group (0.16 ± 0.06) compared to the probing group (0.14 ± 0.06) (*p* = 0.005). This shows the potential of MLR for predicting the risk of recurrence in CNLDO. The analysis of PLR values revealed a statistically significant increase in the recurrence group (95.13 ± 24.34) compared to the probing group (82.23 ± 22.77) (*p* < 0.001). PLR may have the potential to assess the risk of recurrence in CNLDO.

### 3.2. Receiver Operating Characteristic (ROC) Analysis Results (Table 3 and Table 4 and Figure 2)

We conducted ROC analysis to assess the diagnostic performance of inflammatory markers in predicting the recurrence of CNLDO. For the NLR, the AUC was 0.646 (95% CI: 0.563–0.730), showing statistical significance (*p* = 0.001). The optimal cut-off value for the NLR was ≥0.68, with a sensitivity of 83.87% and a specificity of 40.91%. These findings suggest that the NLR may serve as a potential marker for predicting recurrence, although its specificity is limited. The AUC for the MLR was 0.633 (95% CI: 0.549–0.716), which was statistically significant (*p* = 0.002). The optimal cut-off value for MLR was ≥0.12, yielding a sensitivity of 70.97% and a specificity of 53.64%, indicating an intermediate diagnostic performance. The PLR demonstrated an AUC of 0.659 (95% CI: 0.575–0.742), with statistical significance (*p* < 0.001). The optimal cut-off value for PLR was determined to be ≥81.47, with a sensitivity of 70.97% and a specificity of 56.36%, reflecting moderate diagnostic performance. In summary, ROC analysis underscores the potential utility of the NLR, MLR, and PLR as diagnostic markers for predicting recurrence in CNLDO, each exhibiting varying degrees of sensitivity and specificity.

**Figure 2:**
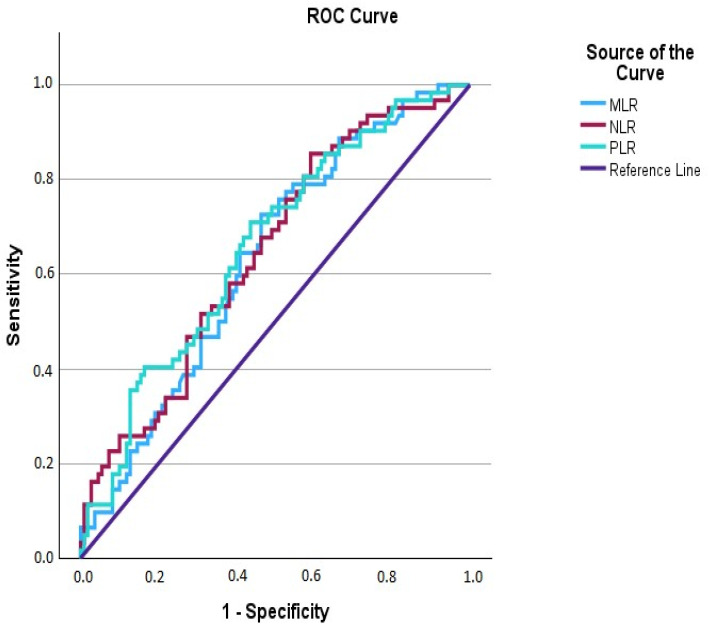
Receiver operating characteristic (ROC) analysis for NLR, MLR, and PLR.

**Table 3:** Receiver operating characteristic (ROC) analysis for NLR, MLR, and PLR.

	*Area*	*95% CI*	*+PV*	*95% CI*	*−PV*	*95% CI*	*p Value*
**NLR**	0.646	0.563–0.730	50.0	41.5–58.5	74.0	67.4–79.7	0.001
**MLR**	0.633	0.549–0.716	41.8	36.7–47.1	74.2	64.1–82.2	0.002
**PLR**	0.659	0.575–0.742	50.0	42.5–57.5	76.7	69.3–82.7	<0.001

CI: asymptomatic 95% confidence interval, PV: predictive value, NLR: neutrophil-to-lymphocyte ratio, MLR: monocyte-to-lymphocyte ratio, PLR: platelet-to-lymphocyte ratio.

**Table 4:** Optimal cut-off values and diagnostic performance of inflammatory biomarkers.

	Cutoff	Sensitivity	95% CI	Specificity	95% CI
**NLR**	>0.68	83.87	72.3–92.0	40.91	31.6–50.7
**MLR**	>0.12	70.97	58.1–81.8	53.64	43.9–63.2
**PLR**	>81.47	70.97	58.1–81.8	56.36	46.6–65.8

CI: asymptomatic 95% confidence interval, NLR: neutrophil-to-lymphocyte ratio, MLR: monocyte-to-lymphocyte ratio, PLR: platelet-to-lymphocyte ratio.

## 4. Discussion

CNLDO is a prevalent condition, affecting approximately 6–20% of newborns. Among the available treatment options, probing achieves a success rate of 80–90%, although recurrence can occur in the remaining cases. This study explores the relationship between post-probing recurrence and systemic inflammatory biomarkers, specifically the NLR, MLR, and PLR in children with CNLDO. Our findings indicate that inflammatory biomarkers based on complete blood cell counts, obtained from pre-probing blood samples, are altered in children who experience recurrence following probing, suggesting that inflammatory processes may play a role in CNLDO recurrence.

Our study observed that the NLR was higher in the recurrence group. Neutrophils play a crucial role in the acute inflammatory response, and the elevated NLR is likely attributable to the activation and proliferation of neutrophils triggered by inflammation following probing [10]. Furthermore, increased neutrophil count and dysfunction may significantly contribute to the infection and inflammation associated with recurrent CNLDO [16]. Specifically, the mean NLR value was 1.03 in the probing group compared to 1.28 in the recurrence group (*p* = 0.011), highlighting the association between systemic inflammation and recurrence.

The monocyte count and MLR were significantly higher in the recurrence group compared to the probing group. Given that monocytes play key roles in inflammation and tissue repair, the elevated MLR may reflect the response of monocytes to the inflammatory processes occurring within the nasolacrimal duct [9,17]. This increase may result from monocyte activation induced by the inflammatory mechanisms involved in the development and progression of recurrent CNLDO [17,18]. Monocytes regulate the inflammatory response and facilitate tissue regeneration within the nasolacrimal duct through cytokine production and phagocytosis.

The platelet count and PLR were also higher in the recurrence group. Since platelets play a critical role in inflammation and tissue repair by secreting various mediators, the elevated PLR may contribute to post-probing inflammation through platelet activation and proliferation [19,20]. Moreover, platelets are involved in tissue repair processes related to inflammation within the nasolacrimal duct [21,22,23]. Platelets promote epithelial cell proliferation and tissue regeneration in the nasolacrimal duct tissue by secreting growth factors.

Our results showed that, while individual counts of neutrophils, monocytes, and platelets did not differ significantly between groups, complete blood cell count-based inflammatory biomarkers were still significantly higher in the recurrence group. This suggests that disruptions in the balance of systemic immune mechanisms may play an important role in the management of CNLDO [24,25]. Inflammation is a key factor in the pathogenesis of CNLDO [10,26]. However, previous studies have demonstrated that the probing procedure used to treat the obstruction can also trigger an inflammatory response, potentially leading to further obstruction [11]. Probing may induce a local inflammatory response through mechanical damage to the nasolacrimal duct epithelium, which can lead to the release of pro-inflammatory cytokines, and infiltration of neutrophils, platelets, and monocytes, resulting in tissue edema.

Our findings align with previous studies. In a recent study, Elibol et al. reported that the Systemic Immune–Inflammation Index (calculated as neutrophil count × platelet count/lymphocyte count) may be a valuable biomarker for identifying cases of CNLDO that are unlikely to resolve spontaneously and require probing [27]. Atum et al. reported increased NLR and MLR values in patients with lacrimal drainage system obstruction, while Chaplin et al. found elevated inflammatory markers in patients who experienced recurrence after probing [4,8]. Similarly, Kashkouli et al. suggested that higher failure rates in delayed probing might be linked to inflammation [1]. The anatomical structure of the nasolacrimal duct may also increase the risk of infection and inflammation by hindering secretion drainage [28,29,30]. Thus, disruption in immune system homeostasis, structural features of the nasolacrimal duct, and the probing procedure itself may trigger inflammatory processes that contribute to recurrence. Our study found that NLR, MLR, and PLR values were significantly higher in the recurrence group, consistent with the literature.

Comorbid inflammatory conditions such as allergic rhinitis [31], chronic tonsillitis [14], and adenoid hypertrophy [32], which are common in patients with CNLDO, can also affect the levels of inflammatory biomarkers. Simsek et al. highlighted that the imbalance in neutrophil and lymphocyte counts in cases of chronic adenoid or adenotonsillar hypertrophy is reflected in the NLR [26]. These findings suggest that inflammatory mechanisms may converge across different nasolacrimal system pathologies [32,33]. In this study, when these comorbid conditions were taken into consideration, no significant differences were observed between the primary probing group and the recurrence group.

Our ROC curve analysis indicated that these inflammation biomarkers have moderate performance in predicting recurrence, with AUC values of 0.72 for the NLR, 0.70 for the MLR, and 0.68 for the PLR. The optimal cut-off points were determined as 1.15 for the NLR, 0.25 for the MLR, and 110 for the PLR, suggesting the moderate discriminative power of these biomarkers. The clinical implications of these findings suggest that complete blood cell count-based biomarkers may be valuable predictive factors in managing CNLDO. Evaluating NLR, MLR, and PLR values before probing could help predict the risk of recurrence, allowing for more personalized patient management. For instance, more invasive treatments, such as tube intubation, may be considered earlier in patients with an elevated inflammatory status [8,24]. Post-probing, systemic, or local anti-inflammatory agents might be used to reduce inflammation. Understanding the factors regulating systemic immunologic homeostasis could also lead to new treatment strategies for the disease [34].

However, our study is not without its limitations, including its retrospective design, relatively small sample size, and single-center nature, which may limit its generalizability. Additionally, the study is limited by the measurement of inflammatory markers at a single time point without serial measurements before and after probing, as well as the absence of a control group of healthy children for comparison.

## 5. Conclusions

In conclusion, our findings underscore the importance of complete blood cell count-based inflammatory biomarkers in managing CNLDO, guiding clinical applications from personalized management to inflammation modulation and new therapeutic approaches. Future studies should further investigate blood parameter changes before and after probing for a more comprehensive understanding. Our findings should be interpreted cautiously and validated through larger, prospective studies to determine the precise sensitivity and specificity of biomarkers in predicting recurrence.

## 6. Summary

Congenital nasolacrimal duct obstruction (CNLDO) is a common condition affecting 6–20% of newborns, with probing achieving an 80–90% success rate, though recurrence is a notable challenge. This study investigates the association between post-probing recurrence and systemic inflammatory biomarkers, specifically the neutrophil-to-lymphocyte ratio (NLR), monocyte-to-lymphocyte ratio (MLR), and platelet-to-lymphocyte ratio (PLR), using pre-probing blood samples. Our findings reveal significant alterations in these inflammatory markers in children with recurrence, indicating that inflammatory processes may influence CNLDO recurrence. Specifically, elevated NLR, MLR, and PLR values were observed in the recurrence group, reflecting the role of neutrophils and monocytes in inflammation and tissue repair. ROC curve analysis demonstrates moderate predictive performance for these biomarkers in forecasting recurrence, suggesting that they could aid in personalized patient management. While the study’s retrospective design and other limitations warrant caution, the results underscore the potential for complete blood cell count-based inflammatory biomarkers to enhance clinical strategies in CNLDO management. Future research should explore changes in these biomarkers before and after probing to further elucidate their role in predicting recurrence.

## Figures and Tables

**Table 1:** Comparison of demographic characteristics between the probing and recurrence groups in patients with congenital nasolacrimal duct obstruction.

			Probing Group			Recurrence Group			
			*Mean*	*SD*	*N*	*Mean*	*SD*	*N*	*p Value*
**Mean Age (months)**		*initial probing*	15.00	4.06	110	ᶜ 15.83	4.02	62	0.199 ^a^
		*recurrence*				ᶜ 37.80	13.34	62	
						*p* < 0.001ᶜ			
**Sex**		**Male**			53			28	0.413 ᵇ
		**Female**			57			34	
**Comorbid situations**	Adenoid and tonsillary hypertrophy	(−)			100			60	0.126 ᵇ
		(+)			10			2	
	Sinusitis	(−)			104			61	0.211 ᵇ
		(+)			6			1	
	Allergic rhinitis	(−)			98			55	0.563 ᵇ
		(+)			12			7	
	Pharyngitis	(−)			108			61	0.705 ᵇ
		(+)			2			1	

SD: standard deviation, N: number, ^a^: independent samples *t*-test, ^b^: Chi-square test, ^c^: paired samples test.

**Table 2:** Comparison of complete blood cell count parameters between the probing and recurrence groups in patients with congenital nasolacrimal duct obstruction.

Probing Group				Recurrence Group			
	*Mean*	*SD*	*Median*	*Mean*	*SD*	*Median*	*p Value*
**White blood cells**	8.81	1.92	9.23	9.36	1.74	9.00	0.064 ^a^
**Lymphocytes**	4.34	0.89	4,65	4.11	0.86	3.63	0.105 ^a^
**Monocytes**	0.68	0.28	0.66	0.71	0.60	0.58	0.698 ^a^
**Neutrophils**	3.96	2.13	3.58	4.16	1.78	3.73	0.524 ^a^
**Eosinophils**	0.26	0.23	0.20	0.24	0.16	0.20	0.589 ^d^
**Basophils**	0.04	0.08	0.03	0.04	0.08	0.03	0.907 ^d^
**Hemoglobin**	11.96	1.59	11.75	14.38	12.93	12.60	0.147 ^a^
**Platelets**	360.8	107.8	352.00	350.9	90.4	349.50	0.540 ^a^
**NLR**	0.86	0.39	0.73	1.12	0.56	1.03	**0.002 ^a^**
**MLR**	0.14	0.06	0.13	0.16	0.06	0.15	**0.005 ^a^**
**PLR**	82.23	22.77	74.65	95.13	24.34	97.97	**<0.001 ^a^**

SD: standard deviation, ^a^: independent samples *t*-test, ^d^: Mann–Whitney U test, **bold**: statistically significant. NLR: neutrophil-to-lymphocyte ratio. MLR: monocyte-to-lymphocyte Ratio. PLR: platelet-to-lymphocyte ratio.

## Data Availability

The original contributions presented in this study are included in the article; further inquiries can be directed to the corresponding author.
